# Redescription and molecular characterization of *Placobdella cryptobranchii* (Johnson & Klemm, 1977) (Glossiphoniidae, Hirudinida)

**DOI:** 10.3897/zookeys.338.5995

**Published:** 2013-10-02

**Authors:** William E. Moser, Jeffrey T. Briggler, Dennis J. Richardson, Chawna D. Schuette, Charlotte I. Hammond, William A. Hopkins, Eric A. Lazo-Wasem

**Affiliations:** 1 Smithsonian Institution, National Museum of Natural History, Department of Invertebrate Zoology, Museum Support Center MRC 534, 4210 Silver Hill Road, Suitland, MD 20746; 2 Missouri Department of Conservation, 2901 W. Truman Blvd, Jefferson City, MO 65109; 3 School of Biological Sciences, Quinnipiac University, 275 Mt. Carmel Avenue, Hamden, Connecticut 06518; 4 Saint Louis Zoo, Herpetology & Aquatics, One Government Drive, St. Louis, MO 63110; 5 Department of Fish and Wildlife Conservation, Virginia Tech, 106 Cheatham Hall, Blacksburg, VA 24061; 6 Division of Invertebrate Zoology, Peabody Museum of Natural History, Yale University, P.O. Box 208118, New Haven, Connecticut 06520

**Keywords:** *Placobdella cryptobranchii*, *Batracobdella*, *Desserobdella*, *Cryptobranchus bishopi*, *Cryptobranchus alleganiensis bishopi*, Ozark Hellbender, Glossiphoniidae, Hirudinea, Rhychobdellida, Clitellata, leech

## Abstract

*Placobdella cryptobranchii* (Johnson & Klemm, 1977) was originally described from specimens collected from Ozark Hellbenders (*Cryptobranchus alleganiensis bishopi*) from the North Fork of the White River in Missouri, U.S.A. Leeches collected during August 2009 to August 2011 from five localities in Missouri (including the type locality) facilitated a redescription and molecular characterization of *Placobdella cryptobranchii*. *Placobdella cryptobranchii* has a rusty, reddish-brown dorsum with 2 lateral rows of unpigmented papillae, two unpigmented nuchal bands, unpigmented patches, and pair of four pre-anal papillae. Molecular comparison of CO-I sequence data from *Placobdella cryptobranchii* revealed a 93–94% similarity to *Placobdella ornata* and 10–17% difference among other species of *Placobdella*.

## Introduction

The hellbender (*Cryptobranchus alleganiensis alleganiensis* and *Cryptobranchus alleganiensis bishopi*) is among the largest salamanders in the world, but is unfortunately imperiled across much of its range in North America. *Batracobdella cryptobranchii* was described by Johnson and Klemm (1977) based upon specimens collected in 1972 by Nickerson and Mays (1973) on Ozark hellbenders (*Cryptobranchus alleganiensis bishopi*) from the North Fork of the White River in Missouri, U.S.A. Reflecting taxonomic instability in a species that had not been reported since its original description, *Batracobdella cryptobranchii* was subsequently transferred to the genus *Actinobdella* by Sawyer (1986), to the genus *Desserobdella* by Barta and Sawyer (1990), and to the genus *Placobdella* by Moser et al. (2006).

In distribution and natural history investigations, *Placobdella cryptobranchii* was reported from additional localities in Arkansas and Missouri (Moser et al. 2006; Moser et al. 2008), but little additional morphological data were added in these accounts. Moser et al. (2008) reported a metameric pattern on the dorsal surface of *Placobdella cryptobranchii*, however, Johnson and Klemm (1977) stated that the dorsum of preserved specimens of *Placobdella cryptobranchii* was smooth and no metameric markings were present. Klemm (1982; 1985) noted that the dorsal surface of some preserved specimens of *Placobdella cryptobranchii* had a metameric pattern. The present study provides a redescription and molecular characterization of *Placobdella cryptobranchii* from its type locality and other localities.

## Materials and methods

### Collection of Leeches

As part of a long term monitoring program of *Cryptobranchus alleganiensis bishopi* populations of by one of the authors (JTB), Ozark hellbenders were captured by hand from 18 August 2009 through 26 August 2011 from the Current River (Carter Co., Ripley Co., and Shannon Co., Missouri), Eleven Point (Oregon Co., Missouri), and the type locality of *Placobdella cryptobranchii*, North Fork of the White River (Ozark Co., Missouri), and examined for leeches. Due to the sensitive status of *Cryptobranchus alleganiensis bishopi*, exact localities are not given. Leeches were also removed from *Cryptobranchus alleganiensis bishopi* specimens collected from the Eleven Point River (Oregon Co., Missouri) and housed at the Saint Louis Zoo for propagation efforts.

Specimens were relaxed, examined, and fixed as described by Moser et al. (2006). Other specimens were maintained alive and mudpuppies (*Necturus maculosus*) were introduced as a potential host and leech activity was observed. For internal anatomy investigations, three specimens were pressed, stained with Semichon’s acetocarmine, and mounted in Canada balsam for examination by light microscopy according to techniques outlined by Richardson (2006), as modified by Richardson and Barger (2006), and two specimens were dissected. Terminology for plane shapes follows Clopton (2004). Specimens were deposited in the Smithsonian Institution, National Museum of Natural History (USNM), Washington, District of Columbia and the Peabody Museum of Natural History (YPM), Yale University, New Haven, Connecticut.

### DNA Analyses

Molecular analyses were conducted on newly collected material according to Richardson et al. (2010) as follows: DNA was isolated from the caudal suckers of individual leeches with the DNeasy Blood & Tissue Kit from Qiagen (Cat. No. 69504), following the protocol given for the purification of total DNA from animal tissues (spin-column). For the proteinase K treatment step, tissue samples were lysed overnight at 56°C. DNA was eluted from the spin columns with 150 µl of buffer.

PCR reactions were prepared using the Illustra PuRe Taq Ready-To-Go PCR beads from GE Health Care (Cat. No. 27-9559-01). Primers were purchased from Invitrogen and were comprised of 2 primers each for cytochrome c oxidase subunit I (CO-I) as specified by Light and Siddall (1999). Specifically the CO-I primers were LCO1490 (5’GGTCAACAAATCATAAAGATATTGG 3’) and HCO2198 (5’TAAACTTCAGGGTGACCAAAAAATCA 3’). Final volume of PCR reactions was 25 µl with 2 µl of leech genomic DNA added per reaction. DNA was amplified under the following PCR conditions: 94°C for 5 min.; 35 cycles of (94°C for 30 sec, 50°C for 30 sec, 72°C for 45 sec); 72°C for 7 min. Following PCR, samples were cleaned using a QIAquick PCR purification kit from Qiagen (Cat. No. 28104).

Purified PCR products were sequenced using the HCO2198 primer and the LCO1490 primer for the Cytochrome c oxidase subunit I products by the W. M. Keck Foundation Biotechnology Resource Laboratory at Yale University. The DNA sequences were aligned using Clustal W version 2 (Larkin et al. 2007) and checked manually using SeaView 4 (Gouy et al. 2010) and then analyzed using PAUP* 4.0b10 (Swofford 2002), deposited in GenBank (http://www.ncbi.nlm.nih.gov/genbank/), and compared to other leech DNA sequences contained within Genbank.

## Results and discussion

**Figure 1.**
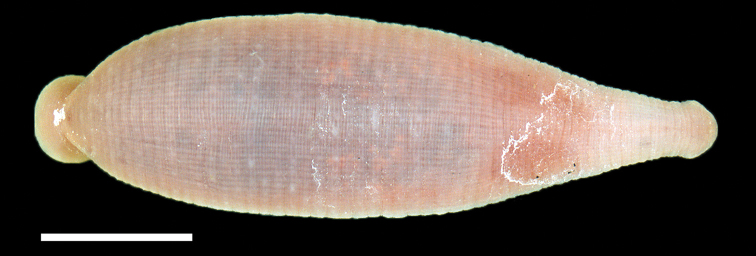
Holotype specimen of *Placobdella cryptobranchii*, USNM 54365, dorsal surface. Scale bar equals 2 mm.

Examination of the type series of *Placobdella cryptobranchii* included the Holotype (USNM 54365) and Paratypes (USNM 54366, 10 specimens; MPM 2675-2677). All of the specimens in the type series were collected on 24 September, 1972 from the North Fork of the White River in Ozark County, Missouri, U.S.A. The USNM Holotype and Paratype lots are currently stored in 70% ethanol, however, the museum label in the vial of the Holotype indicates that it was preserved in weak formalin (4% formalin), stained in borax carmine, and cleared and stored in methyl salicylate (Figure 1). The Holotype still has a faint wintergreen odor of methyl salicylate. The museum label in the vial of the Paratype indicates that the specimens were preserved and stored in weak formalin (4% formalin).

**Figure 2.**
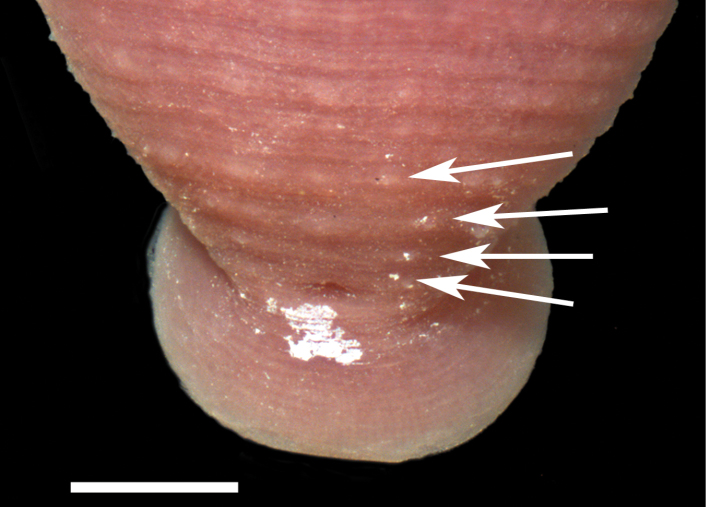
Holotype specimen of *Placobdella cryptobranchii*, USNM 54365, pre-anal papillae (arrows). Scale bar equals 0.5 mm.

Examination of the Holotype and Paratype specimens revealed a narrowly rhomboid body, caudal sucker on a short pedicel, 2 annuli between the male and female gonopores, mouthpore on the rim/lip of the oral sucker, and a faint outline of two pair of eye spots. The dorsal surface of the Holotype and a few Paratypes had minute papillae. Beginning adjacent to the anus and commencing anteriad on either side of the anus in the Holotype and a few Paratypes were two rows of four papillae (the last row – most anteriad – papillae are medially indented) (Figure 2). Any trace of pigmentation or pigmentation pattern has faded.

The following redescription of *Placobdella cryptobranchii* is based on the Holotype (USNM 54365), Paratypes (USNM 54366, 10 specimens), and newly collected specimens consistent with the description of *Batracobdella cryptobranchii* by Johnson and Klemm (1977) from the Current River (Carter Co., Ripley Co., and Shannon Co., Missouri) (USNM 1223071–1223080), Eleven Point River (Oregon Co., Missouri) (USNM 1223081–1223082), Eleven Point River (Oregon Co., Missouri) at the Saint Louis Zoo (USNM 1223083–1223084; USNM 1223088–1223090, three whole mount slides; YPM IZ 06339–06340), and the type locality of *Placobdella cryptobranchii*, North Fork of the White River (Ozark Co., Missouri) (USNM 1223085–1223087).

### Placobdella cryptobranchii

(Johnson & Klemm, 1977) Moser et al., 2006

http://species-id.net/wiki/Placobdella_cryptobranchii

Figures 3 – 5

Batracobdella cryptobranchii Syn. Johnson & Klemm, 1977; *Actinobdella cryptobranchii* Sawyer, 1986; *Desserobdella cryptobranchii* Barta & Sawyer, 1990

#### External morphology.

**Figure 3.**
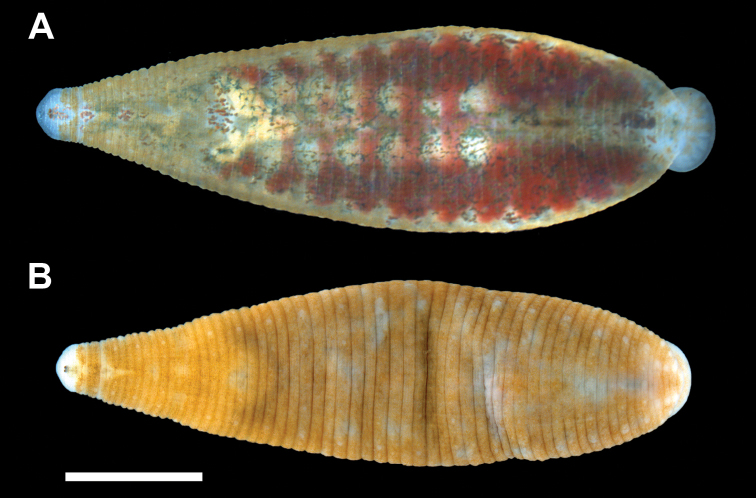
Dorsal surface of *Placobdella cryptobranchii*. **A** Living, YPM IZ 06339 **B** Preserved, YPM IZ 06340. Scale bar equals 2 mm.

**Figure 4.**
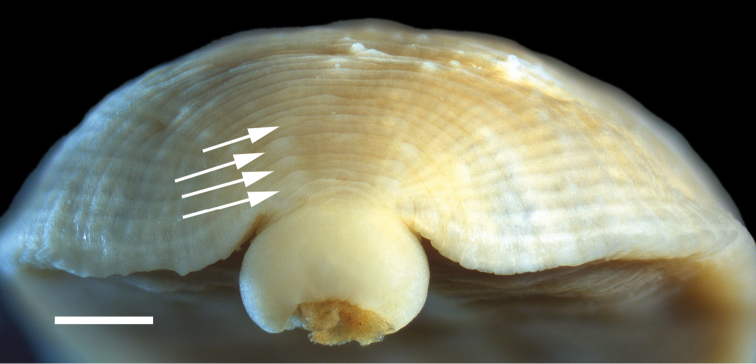
Pre-anal papillae of *Placobdella cryptobranchii* (USNM 1223081). Scale bar equals 0.5 mm.

Body very deeply ovoid to obovoid. Length of preserved specimens 3.6–13.3 mm long, mean±SE: 6.6±0.3 mm (n=42), width at widest point (in posterior half of body) 2.1–6.6 mm, mean±SE: 3.8±0.2 mm (n=42). Dorsum rusty, reddish-brown with 2 lateral rows of unpigmented papillae (Figure 3); smaller sensillae on every annulus (absent on poorly-preserved specimens). Apical cephalic region unpigmented, extending and tapering posteriorly through two thin nuchal bands (Figure 3). Two pair of eye spots (one pair much larger than the other) within cephalic unpigmented region. Unpigmented genital bar and anal patch with some specimens possessing unpigmented patch in between unpigmented genital bar and anal patch (Figure 3). Pigmentation gradually fades in ethanol and may not be present in poorly-preserved specimens. Beginning adjacent to the anus, just anterior to the anus furrow, and commencing anteriad are two rows of 4 pre-anal papillae (the last row, most anteriad, papillae are medially indented) (Figure 4). Caudal sucker small, 0.4–1.9 mm in diameter, mean±SE: 1.1±0.1 (n=35), and unpigmented or with large unpigmented patches. No papillae on caudal sucker or 1 row of small papillae on the lateral edge. Ventrum unpigmented with male and female gonopores in furrows and separated by 2 annuli.

#### Alimentary tract.

**Figure 5.**
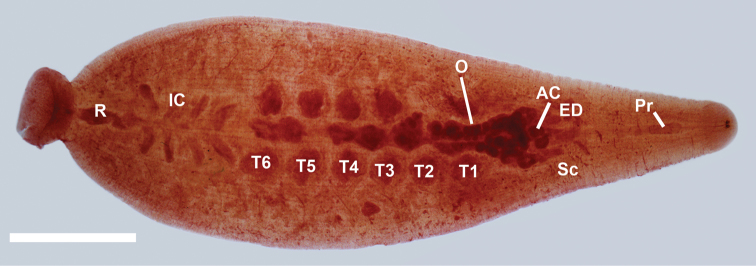
Internal anatomy of *Placobdella cryptobranchii* (USNM 1223088), ventral view, atrial cornuae (**AC**), ejaculatory duct (**ED**), intestinal ceca (**IC**), ovisac (**O**), proboscis (**Pr**), rectum (**R**),salivary cells (**Sc**), testisac (**T1–T6**). Scale bar equals 2 mm.

Proboscis pore just posteriad of the rim/lip of the oral sucker. Blunt-tipped proboscis, nearly uniformly cylindrical, slightly enlarged at base, and in membranous sheath (Figure 5). In the anterior third of the leech, salivary cells strewn on either side of the proboscis (Figure 5). Salivary cells most numerous in three somites at the base of the proboscis, and more scattered anteriad and posteriad of that region. Retractor muscle attached to dorsal body wall and joining salivary ductule bundles attaching at each side of the base of the proboscis. Slim, flaccid esophagus extends from the base of the proboscis with one pair sac-like mycetomes [called esophageal diverticulum by Johnson and Klemm (1977)]. Seven pair of diverticulated crop ceca, last pair extending posteriorly and diverticulated into four sections. Four pair of simple, saccular intestinal ceca with last pair extending posteriad. Simple rectum opening to anus, located one annulus anteriad of the caudal sucker.

#### Reproductive system.

(Male) Male gonopores slightly raised. Male atrium opening into paired very broadly orbicular atrial cornuae extending laterally and anteriorly from male gonopore into robust, coiled, muscular ejaculatory ducts, recurving posteriorly to robust seminal vesicles and narrow vas deferentia connecting to testisacs (Figure 5). Six pair of testisacs, each testisac located in the space between a pair of crop ceca (Figure 5). (Female) Female gonopore simple, opening to pair of bifurcated ovisacs and located within coelomic space that is attached on the ventral body wall (Figure 5). Ovisac length depends on the reproductive condition of the leech. In the specimens examined in this study, the ovisac extended posteriad to the sixth testisac or past the sixth testisac. Anterior, cecum-like extensions of the ovisacs are smaller and more delicate than those of the main posterior section.

##### Laboratory observations on life history

6 May, 2013: Presented *Placobdella cryptobranchii* with brood to *Necturus maculosus* in 5 gallon tank. No reaction; 2 others no reaction after one hour; 2 others left over-night–did not respond.

4 June, 2013: Presented 2 *Placobdella cryptobranchii* to *Necturus maculosus* in 5 gal tank. – one did not respond and one exhibited vigorous host seeking behavior.: Leech examined skin of *Necturus maculosus* for about first 10 minutes but did not feed. Host seeking behavior subsided over the next few moments and the leech then exhibited no further interest.

## Remarks

Johnson and Klemm (1977) found no evidence of dorsal color pattern or metameric markings on preserved specimens of *Placobdella cryptobranchii*, however, Klemm (1982; 1985) mentioned that some preserved specimens have a metameric pattern. Moser et al. (2008) described a dorsal pigmentation pattern in *Placobdella cryptobranchii* and this pattern was further clarified in the current study along with a description of dorsal papillae patterns. This discrepancy is easily explained since the leech pigmentation pattern fades in ethanol (Moser et al. 2008) and is not retained in poorly preserved specimens.

Intraspecific comparison of 639 nucleotides of CO-I revealed differences of 0.3% to 3.3% (2–21 nucleotides) among seven specimens of *Placobdella cryptobranchii* (GenBank KF601755–KF601761) collected from the Current River (Carter Co., Ripley Co., and Shannon Co., Missouri), Eleven Point (Oregon Co., Missouri), and the type locality of *Placobdella cryptobranchii*, North Fork of the White River (Ozark Co., Missouri). In contrast, CO-I sequence data among seven specimens of *Placobdella cryptobranchii* revealed interspecific differences of 5.8% to 6.8% (37–43 nucleotides) when compared to five specimens of *Placobdella ornata* (Verrill, 1872) (GenBank JQ8128–JQ8132) collected from the type locality (West River, New Haven County, Connecticut), differences of 6.3% to 7.1% (40–45 nucleotides) among four specimens of *Placobdella ornata* collected from the type locality (Shivericks Pond, Falmouth, Barnstable County, Massachusetts) of *Placobdella phalera* (Graf, 1899) (junior synonym of *Placobdella ornata*) (GenBank JQ812133–JQ812136), differences of 10.4% to 12.1% (66–77 nucleotides) among two specimens of *Placobdella translucens* Sawyer and Shelley, 1976 (GenBank AY047328, JX122778), differences of 14.8% to 15.4% (94–98 nucleotides) from 1 specimen of *Placobdella picta* (Verrill, 1872) (GenBank AF116020), and differences of 16.2% to 16.9% (103–109 nucleotides) from 1 specimen of *Placobdella biannulata* (Moore, 1900) (GenBank AF116021).

*Placobdella cryptobranchii* is morphologically and molecularly similar to *Placobdella ornata* as described by Verrill (1872) and redescribed by Moser et al. (2012). However, *Placobdella cryptobranchii* has two lateral rows of white-tipped papillae and *Placobdella ornata* has three rows of papillae (a medial and two lateral rows) with black-tipped papillae on the medial row and a medial pigment stripe. *Placobdella ornata* has two rows of five pre-anal papillae and *Placobdella cryptobranchii* has two rows of four pre-anal papillae and the last, most anteriad row, has papillae that are medially indented. *Placobdella cryptobranchii* is distributed in the Ozark Highlands region of Arkansas and Missouri, and the Ozark Hellbender (*Cryptobranchus alleganiensis bishopi*) and mudpuppy (*Necturus maculosus*) have been reported as hosts (Johnson and Klemm 1977; Moser et al. 2006; Briggler and Moser 2008; Moser et al. 2008). *Placobdella ornata* is currently known only from New England (Moser et al. 2012). The only host record for *Placobdella ornata* is the common musk turtle (*Sternotherus odoratus*) (Graf 1899) and this report is tenuous as no voucher specimens were deposited. Although closely related, *Placobdella cryptobranchii* is morphologically and molecularly distinct from *Placobdella ornata*.

Based on the life history experiments, it was concluded that *Placobdella cryptobranchii* does not utilize *Necturus maculosus* as a normal host, suggesting that the occurrence of four *Placobdella cryptobranchii* on a Red River mudpuppy from the Eleven Point River in Missouri by Briggler and Moser (2008) may have been an isolated event. Until further data are available, *Cryptobranchus alleganiensis bishopi* should be considered the only primary host for *Placobdella cryptobranchii*, which appears to exhibit a fairly high level of host specificity. The inability to induce feeding of *Placobdella cryptobranchii* on *Necturus maculosus* in the laboratory suggests that any further decline of *Cryptobranchus alleganiensis bishopi* will place *Placobdella cryptobranchii* in danger of extinction.

## Supplementary Material

XML Treatment for Placobdella cryptobranchii
